# A Bivalent Live-Attenuated Vaccine for the Prevention of Equine Influenza Virus

**DOI:** 10.3390/v11100933

**Published:** 2019-10-11

**Authors:** Pilar Blanco-Lobo, Laura Rodriguez, Stephanie Reedy, Fatai S. Oladunni, Aitor Nogales, Pablo R. Murcia, Thomas M. Chambers, Luis Martinez-Sobrido

**Affiliations:** 1 Department of Microbiology and Immunology, University of Rochester, Rochester, NY 14642, USA; piblanlo@gmail.com (P.B.-L.); laurita85oviedo@hotmail.com (L.R.); nogales.aitor@inia.es (A.N.); 2 Agencia Española de Medicamentos y Productos Sanitarios, E28022 Madrid, Spain; 3 Department of Veterinary Science, Gluck Equine Research Center, University of Kentucky, Lexington, KY 40546, USA; sereed0@uky.edu (S.R.); kanmi01@gmail.com (F.S.O.); Thomas.Chambers@uky.edu (T.M.C.); 4 Center for Animal Health Research- National Institute for Agricultural and Food Research and Technology, Valdeolmos, 28130 Madrid, Spain; 5 MRC-University of Glasgow Centre for Virus Research, Glasgow G61 1AF, UK; Pablo.Murcia@glasgow.ac.uk

**Keywords:** equine influenza virus, live-attenuated influenza vaccine, master donor virus, reverse genetics techniques, recombinant virus

## Abstract

Vaccination remains the most effective approach for preventing and controlling equine influenza virus (EIV) in horses. However, the ongoing evolution of EIV has increased the genetic and antigenic differences between currently available vaccines and circulating strains, resulting in suboptimal vaccine efficacy. As recommended by the World Organization for Animal Health (OIE), the inclusion of representative strains from clade 1 and clade 2 Florida sublineages of EIV in vaccines may maximize the protection against presently circulating viral strains. In this study, we used reverse genetics technologies to generate a bivalent EIV live-attenuated influenza vaccine (LAIV). We combined our previously described clade 1 EIV LAIV A/equine/Ohio/2003 H3N8 (Ohio/03 LAIV) with a newly generated clade 2 EIV LAIV that contains the six internal genes of Ohio/03 LAIV and the HA and NA of A/equine/Richmond/1/2007 H3N8 (Rich/07 LAIV). The safety profile, immunogenicity, and protection efficacy of this bivalent EIV LAIV was tested in the natural host, horses. Vaccination of horses with the bivalent EIV LAIV, following a prime-boost regimen, was safe and able to confer protection against challenge with clade 1 (A/equine/Kentucky/2014 H3N8) and clade 2 (A/equine/Richmond/2007) wild-type (WT) EIVs, as evidenced by a reduction of clinical signs, fever, and virus excretion. This is the first description of a bivalent LAIV for the prevention of EIV in horses that follows OIE recommendations. In addition, since our bivalent EIV LAIV is based on the use of reverse genetics approaches, our results demonstrate the feasibility of using the backbone of clade 1 Ohio/03 LAIV as a master donor virus (MDV) for the production and rapid update of LAIVs for the control and protection against other EIV strains of epidemiological relevance to horses.

## 1. Introduction

Equine influenza is an upper respiratory disease characterized by fever, lethargy, coughing, dyspnea, and nasal discharge that affects horses and other equids and has a severe impact on the equine industry [1]. The causative agent, equine influenza virus (EIV), is a member of the *Orthomyxoviridae* family of negative-stranded RNA viruses with a segmented genome [2]. The first EIV isolated in Europe in 1956 (A/equine/Prague/1956) was an influenza A virus (IAV) H7N7 subtype that is believed to have disappeared from the equine population [3]. H3N8 EIV was initially isolated in 1963 in the United States (US) and became widely spread causing major outbreaks around the world, which persist today [2,4,5,6]. At the end of the 1980s and as a result of antigenic drift, H3N8 EIV diverged into two antigenically distinct Eurasian and American lineages, named according to the geographic origin of the isolates [7,8]. EIVs from the Eurasian lineage have not been detected since 2005 [9,10]. The American lineage subsequently evolved into South-American, Kentucky, and Florida sublineages [11]. Further evolution of the Florida sublineage resulted in the emergence of two groups of EIVs classified on the basis of the HA sequence: Clade 1 and clade 2 [12,13,14]. Currently, clade 1 EIVs are predominantly found in the US whereas clade 2 EIVs are primarily circulating in Europe and Asia [2,15,16,17,18,19]. EIVs from the clade 1 Florida sublineage have caused outbreaks in other parts of the world [5,20,21,22,23,24,25] and a clade 2 EIV was detected in a horse in California that was newly imported from Europe [26]. Therefore, both clades of the Florida sublineage of EIVs are currently co-circulating and co-evolving worldwide. Because of this phenomenon, and due to the frequent international transport of horses, the World Organization for Animal Health (OIE, Office International des Epizooties) recommends including representative viruses from both clade 1 and clade 2 of the Florida sublineage in the composition of H3N8 EIV vaccines [27].

Prevention and control of H3N8 EIV in the equine population rely on hygiene [28], quarantine [29], and vaccination programs [30] to reduce infection and spread between horses. A large number of vaccine strategies to control H3N8 EIV in horses are available [31,32]: (1) Inactivated influenza vaccines (IIV), (2) subunit vaccines, (3) DNA vaccines, (4) viral-vector vaccines, and (5) a live attenuated influenza vaccine (LAIV) [33,34,35,36,37,38]. However, only few IIVs include both clade 1 and clade 2 strains of the Florida sublineage in their composition [27,39], as recommended by the OIE. Numerous reports have evaluated the efficacy of one or more EIV vaccines in horses [34,35,40,41,42,43,44,45,46]. In general, protective immunity induced by intramuscular IIV relies on the induction of neutralizing antibodies with a weak induction of cellular responses, limiting cross-protection [31,46,47,48]. On the other hand, intranasal LAIV administration mimics the natural route of infection and is able to induce long-lasting immune adaptive humoral and cellular responses. Therefore, LAIVs provide better protection than their IIV counterparts [49,50,51]. Only one LAIV is currently commercialized for the prevention of H3N8 EIV and is sold under the name of Flu Avert I.N. (Merck). Flu Avert I.N. contains an attenuated (att), cold-adapted (ca), and temperature-sensitive (ts) A/equine/Kentucky/1/1991 H3N8 generated by serial passage in embryonated chicken eggs at gradually reduced temperatures [52,53]. Flu Avert I.N. has shown an excellent safety profile characterized by low transmission rates to unvaccinated horses and the absence of side-effects after immunization, including in immunocompromised animals [53,54]. In addition, Flu Avert I.N. has been shown to induce protection against homologous and heterologous H3N8 EIVs, probably due to the induction of cross-protective T cell responses [54,55]. Some comparative studies showed that Flu Avert I.N. offers better protection against EIVs than IIVs [4,27,33]. Even though previous studies highlight the benefits of using LAIV to prevent and control EIV infections, the current concern is that Flu Avert I.N. has not been updated since its original commercialization. The mismatch between the H3N8 EIV in Flu Avert I.N. (A/equine/Kentucky/1/1991) and contemporary strains probably explains the reduced vaccine efficacy and the only partial protection against currently circulating viruses among horses vaccinated with Flu Avert I.N. [4,31,56,57]. This is further evidenced by the 2018–2019 outbreaks of EIV in both the US and Europe, involving vaccinated horses [58,59,60]. Moreover, the amino acid changes responsible for the att, ca, and ts phenotype of Flu Avert I.N. have not yet been mapped or characterized.

We have recently described the generation, using reverse genetics techniques, of an EIV LAIV derived from the clade 1 A/equine/Ohio/1/2003 H3N8 (Ohio/03) for the control of EIV infections [61]. This Ohio/03 LAIV was obtained by introducing the mutations responsible for the ts, ca, and att phenotype of the master donor virus (MDV) A/Ann Arbor/6/60 H2N2, an LAIV commercialized in the US for the control of human infections (FluMist, Medimmune) [62]. Importantly, our Ohio/03 LAIV was safe and able to induce protective immune responses in a mouse model of influenza infection against challenge with clade 1 H3N8 wild-type (WT) EIV [61]. Its safety, immunogenicity, and protective profile was also shown in horses, its natural host, suggesting the feasibility of implementing this new EIV LAIV for the control of EIV in the horse population [61]. However, since this new LAIV only contains the clade 1 EIV Ohio/03, it does not follow the current recommendation of the OIE for a vaccine to control EIV in horses [63]. Here, we developed a bivalent EIV LAIV for the protection against clade 1 and clade 2 EIVs of the Florida sublineage. We generated a recombinant virus containing the internal genes of Ohio/03 LAIV (clade 1) [61] and the HA and NA of A/equine/Richmond/1/2007 H3N8 (Rich/07 LAIV) as representative of clade 2. Importantly, we demonstrate the safety and protection efficacy of our bivalent EIV LAIV based on a blend of clade 1 and clade 2 attenuated viruses for its implementation for the protection from EIV in horses.

## 2. Material and Methods

### 2.1. Cells and Viruses

Human embryonic kidney 293T (HEK293T; ATCC CRL-11268) and Madin-Darby canine kidney (MDCK, ATCC CCL-34) cells were cultured at 37 °C with 5% CO_2_ in Dulbecco’s modified Eagle’s medium (DMEM; Mediatech, Inc., Manassas, VA, USA), supplemented with 10% fetal bovine serum (FBS), and 1% PSG (penicillin, 100 units/mL; streptomycin, 100 μg/mL; l-glutamine, 2 mM) [61,64].

Recombinant WT (Ohio/03 WT) and LAIV (Ohio/03 LAIV) EIVs derived from A/equine/Ohio/1/2003 H3N8 were generated by plasmid-based reverse techniques as previously described [61]. The recombinant Rich/07 LAIV was generated by reverse genetics techniques using plasmids encoding the six internal genes (PB2, PB1, PA, NP, M, and NS) of Ohio/03 LAIV [61] and the HA and NA viral segments of A/equine/Richmond/1/2007 H3N8 (Rich/07). WT A/equine/Kentucky/2014 H3N8 (KY/14 WT) and Rich/07 (kindly provided by Dr. Debra Elton at the Animal Health Trust, United Kingdom) were used for challenge experiments. KY/14 WT is a Florida clade 1 heterologous strain yet antigenically similar to our EIV LAIV. We used KY/14 WT instead of Ohio/03 WT for challenge experiments to directly assess protection against a heterologous virus strain. EIVs used in horse challenge experiments were grown in chicken embryonated eggs at 35 °C. Virus titers were determined by 50% egg infectious dose (EID_50_). For viral infections in horses, virus preparations were diluted in phosphate buffered saline (PBS) containing 0.3% bovine serum albumin (BSA) and 1% penicillin and streptomycin (PBS/BSA/PS) to prevent potential bacterial contamination. Stocks of LAIVs were produced in MDCK cells as previously described [61]. Briefly, MDCK cells were infected (MOI 0.01) with the EIV LAIVs. Viral adsorption was carried out at room temperature (RT) for 1 h and cells were subsequently maintained in post-infection DMEM supplemented with 0.3% BSA, 1% PSG, and 1 μg/mL N-tosyl-L-phenylalanine chloromethyl ketone (TPCK)-treated trypsin (Sigma, St. Louis, MO, USA). Viruses were propagated in MDCK cells at 33 °C [65] and viral titers were determined by immunofocus assay (focus forming units/mL, FFU/mL) using the monoclonal antibody (MAb) HB-65 against the viral nucleoprotein (NP) (ATCC HB-65, HL16-L10-4R5) as previously described [66].

### 2.2. Virus Rescue

The Ohio/03 WT and LAIV were generated as previously described [61,66]. For the rescue of Ohio/03 LAIV, ambisense pDZ PB2 and PB1 plasmids containing the LAIV mutations (PB2 N265S; and PB1 K391E, E581G, and A661T) were used along with the remaining pDZ plasmids encoding Ohio/03 WT genes (PA, HA, NP, NA, M, and NS). For the generation of Rich/07 LAIV, the HA (GenBank Accession number: FJ195395.3) and NA (GenBank Accession number: KF559336.1) genes of Rich/07 WT were synthesized de novo and cloned in a pUC57 plasmid (Bio Basic, Amherst, NY, USA). The HA and NA viral segments were then subcloned into the ambisense pDZ plasmid for viral rescue [65,66,67]. For rescue of Rich/07 LAIV, a co-culture (1:1) of HEK293T and MDCK cells (6-well plate format, 1 × 10^6^ cells/well, triplicates) were co-transfected with 1 μg of ambisense pDZ plasmids encoding the six internal genes of Ohio/03 LAIV (pDZ-PB2 LAIV, -PB1 LAIV, -PA, -NP, -M, and -NS) along with the pDZ plasmids encoding the HA and NA (pDZ-HA and -NA) genes of Rich/07 WT. At 12 h post-transfection, the medium was replaced with DMEM supplemented with 0.3% BSA, 1% PSG, and 0.5 μg/mL TPCK-treated trypsin, and incubated at 33 °C. At 3–4 days post-infection (p.i.), viruses present in the tissue culture supernatant (TCS) were collected and used to infect fresh monolayers of MDCK cells (12-well plate format, 5 × 10^5^ cells/well) that were incubated at 33 °C until complete cytopathic effect was observed. Recombinant viruses were plaque-purified and propagated in MDCK cells at 33 °C [68].

### 2.3. RNA Isolation, RT-PCR, and cDNA Digestions

MDCK cells (6-well plate format, 1 × 10^6^ cells/well, triplicates) were infected with Ohio/03 or Rich/07 LAIVs at a multiplicity of infection (MOI) of 0.1 and incubated at 33 °C. At 48 h p.i., total RNA was isolated using TRIzol reagent (Invitrogen, Carlsbad, CA, USA) according to the manufacturer’s specifications. SuperScript^®^ II Reverse Transcriptase (Invitrogen) was used to synthesize complementary (c)DNA that was subsequently used as a template in the PCR. The HA and NA segments of Ohio/03 and Rich/07 LAIVs were amplified using specific primers for HA (Forward5′-AGCAAAAGCAGGGGATATTTCTGTC-3′; Reverse 5′- AGTAGAAACAAGGGTGTTTTTAAC-3′) and NA (Forward5′-AGCAAAAGCAGGAGTTTAAAATG-3′; Reverse 5′-AGTAGAAACAAGGAGTTTTTTCGTAAATTAC-3′). PCR amplified HA segments were digested with Sac I or Aat II and NA segments with Bgl II or Hind III for 1 h at 37 °C. Undigested and digested products were separated by electrophoresis in an agarose gel (1% *w*/*v*) and visualized using an ultraviolet (UV) transilluminator. RT-PCR amplified HA and NA cDNA were confirmed by sequencing (ACGT).

### 2.4. Virus Growth Kinetics

Multicycle growth kinetics were performed by infecting confluent monolayers of MDCK cells (12-well plate format, 5 × 10^5^ cells/well, triplicates) with Ohio/03 WT, Ohio/03 LAIV, or Rich/07 LAIV at a MOI of 0.001. After 1 h viral adsorption at RT, fresh post-infection media was added and cells were incubated at the indicated temperatures (33 °C, 37 °C, or 39 °C). At the specified times p.i., viral titers in TCS were determined by immunofocus assay (FFU/mL) in MDCK cells [66]. The mean values and standard deviations (SDs) were calculated using Microsoft Excel software.

### 2.5. Plaque Assays

Confluent MDCK cell monolayers (6-well plate format, 1 × 10^6^ cells/well) were infected with Ohio/03 WT, Ohio/03 LAIV, or Rich/07 LAIV. After 1 h viral adsorption at RT, cells were overlaid with agar (0.6 % *w*/*v*) and incubated at 33 °C, 37 °C, or 39 °C. At three days p.i., cells were fixed for 1 h at RT with 4% paraformaldehyde (PFA) in PBS and overlays were carefully removed. Fixed cells were then permeabilized (0.5% Triton X-100 in PBS for 15 min at RT) and subjected to a blocking step (2.5% BSA in PBS for 1 h at RT). For immunostaining, the anti-NP MAb HB-65 and vector kits (Vectastain ABC vector kits and DAB HRP substrate kit; Vector, Burlingame, CA, USA) were used according to the manufacturer’s specifications [64,69].

### 2.6. Horse Experiments

Seronegative EIV H3N8 horses (*n* = 18) of mixed sex, aged 1–2 years were used. They were raised at the University of Kentucky’s Maine Chance Farm as part of a dedicated research herd. Horse experiments were approved by the University of Kentucky’s Institutional Animal Care and Use Committee (IACUC, Protocol no. 2007-0153). Horses were assigned into group 1 (*n* = 6) and group 2 (*n* = 6), each containing 3 males and 3 females (pastured separately). They were vaccinated (V1) by aerosol inhalation of a virus preparation containing 3 × 10^8^ FFU of each component of the bivalent EIV LAIV (Ohio/03 and Rich/07 LAIVs) using the Flexineb II portable equine nebulizer/facemask (Flexineb^®^ North America, Union City TN). A booster vaccination (V2) was performed using the same dose and method 29 days after priming. Vaccinations of Groups 1 and 2 were staggered by 2 weeks to avoid cross-contamination and facilitate their separate experimental challenges at 28 days following V2. Additional influenza seronegative horses (*n* = 6, 3 males and 3 females) were used as unvaccinated controls and pastured separately from the vaccinated horses to avoid viral transmission. To evaluate the safety profile of the bivalent EIV LAIV, vaccinated horses were closely monitored from the day of vaccination (day 0) to day 7 post-inoculation of V1 and V2. Clinical signs (recorded by the same observer), rectal temperatures, and virus titers in nasopharyngeal swabs were determined as previously described [61,70]. To evaluate the immunogenicity of the bivalent EIV LAIV, blood samples were collected on the days of vaccination (both V1 and V2) and the day of challenge (day 57). Presence of serum antibodies was measured by hemagglutination inhibition (HAI) assay. Additionally, for group 1 only, 2 additional influenza-seronegative horses (1 male, 1 female) were co-pastured with the vaccinated starting the day after V1, to assess transmission of the LAIV to unvaccinated contacts.

For challenge experiments, vaccinated horses in group 1 and, 2 weeks later, group 2 (*n* = 6) were brought into a BSL-2 isolation barn to be challenged with the heterologous Florida clade 1 KY/14 WT or a homologous Florida clade 2 Rich/07 WT, respectively. Unvaccinated control horses (*n* = 3 for each challenge) were challenged alongside the vaccinated, and the 2 sentinels were challenged alongside group 1. Challenge viruses were administered by aerosol inhalation using the Flexineb II device, with a delivered dose of 5 × 10^7^ EID_50_ units per horse [61,71,72]. This challenge dose was similar to that used in our previous, and other authors’, studies [55,61,73]. To evaluate the protection efficacy of the bivalent EIV LAIV, both vaccinated and unvaccinated control horses were observed daily for clinical signs and rectal temperatures and nasopharyngeal swabs were collected and evaluated from the day of challenge (day 0) through day 8 post-challenge. Swabs were initially tested for non-quantitative viral presence by injection into embryonated eggs (100 μL/egg; triplicate) as previously described [54]. Viral content in nasopharyngeal swabs was determined by immunofocus assay (FFU/mL) in MDCK cells [66]. Virus content was also measured in day + 2 swabs by EID_50_ titration. At 3 days after egg inoculation, allantoic fluid was harvested and the presence of virus was assessed by hemagglutination assay (HA). Samples were considered positive when infection occurred in at least one out of three eggs.

### 2.7. Hemagglutination Inhibition (HAI) Assay

Humoral responses were evaluated pre- (day 0) and post-vaccination (day 28) and pre- (day 0) and post-challenge (7, 14, and 21 days) by HAI assay using Ohio/03, KY/14 and Rich/07 WT viruses as previously described [74]. Sera samples were pre-treated with trypsin/periodate as described [74] to avoid non-specific inhibitors of hemagglutination.

### 2.8. Clinical Monitoring

For assessment of the safety profile of the bivalent EIV LAIV, horses received physical examinations on days 0, 2, 3, and 7 after both prime and boost doses. Likewise, challenged horses received daily physical examinations for 10 days after virus challenge. Clinical examinations included measurement of rectal temperature, heart rate, respiratory rate, auscultation of lung and gut sounds, palpation of submandibular and parotid lymph nodes, presence of nasal discharge, spontaneous coughing, and signs of depression or anorexia. Clinical scoring was calculated based on those clinical signs in which significant changes were observed, as previously described [38] (Table 1). In accordance with the IACUC protocol, horses exhibiting clinical signs were examined by an independent veterinarian who made all decisions regarding treatment and also administered those treatments. This veterinarian was kept blinded regarding the vaccination status of the horses. Clinically affected horses continued to be monitored until clinical signs disappeared. All horses returned to pasture at day + 14 post challenge.

### 2.9. Statistical Analysis

Statistical analysis was performed using the statistical package SPSS version 20.0. In vivo and in vitro studies were analyzed using two-tailed Student’s test. Correlation between HAI and clinical score was analyzed by Pearson correlation test. *p*-values < 0.05 were considered statistically significant.

## 3. Results

### 3.1. Generation and Characterization of Rich/07 LAIV

**Figure 1:**
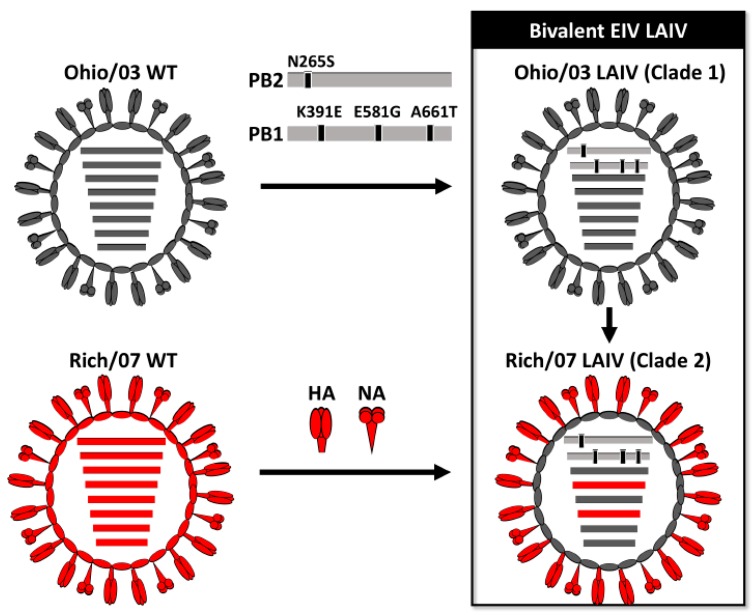
Schematic representation of the bivalent equine influenza virus (EIV) live-attenuated influenza vaccine (LAIV): To generate the clade 1 A/equine/Ohio/1/2003 H3N8 LAIV (Ohio/03 LAIV; top right), the temperature sensitive (ts), cold adapted (ca), and attenuated (att) mutations of the human A/Ann Arbor/6/60 H2N2 LAIV were introduced into the PB2 (N265S) and PB1 (K391E, E581G, and A661T) segments of A/equine/Ohio/1/2003 H3N8 wild-type (WT) (Ohio/03 WT; top left). Ohio/03 LAIV was used as a master donor virus (MDV) to generate clade 2 A/Richmond/1/2007 H3N8 LAIV (Rich/07 LAIV; bottom right), containing the six internal genes (PB2, PB1, PA, NP, M, and NS) from Ohio/03 LAIV and the hemagglutination assay (HA) and neuraminidase (NA) (red) of A/Equine/Richmond/1/2007 H3N8 WT (Rich/07 WT; bottom left). The bivalent EIV LAIV is made by combining Ohio/03 and Rich/07 monovalent LAIVs.

During the last 10 years, the OIE has recommended that commercial EIV vaccines should include representative strains from both the clade 1 and clade 2 Florida sublineage EIVs [9]. A LAIV for the treatment of EIV based on A/equine/Kentucky/1/1991 H3N8 has been commercially available for the treatment of EIV infections in horses but does not fulfill the OIE recommendations. We have recently described an EIV LAIV based on a clade 1 EIV LAIV Ohio/03 [61]. In order to follow the OIE recommendations, we sought to develop a bivalent EIV LAIV containing representative strains of both clade 1 and clade 2 Florida sublineage EIVs. To that end, we generated a recombinant virus containing the internal genes of Ohio/03 LAIV and the HA and NA glycoproteins of Rich/07 H3N8 to develop a clade 2 Rich/07 LAIV. The bivalent EIV LAIV was next generated by blending the Ohio/03 and Rich/07 LAIVs as representatives of the Florida sublineages clade 1 and clade 2 EIV, respectively (Figure 1).

**Figure 2:**
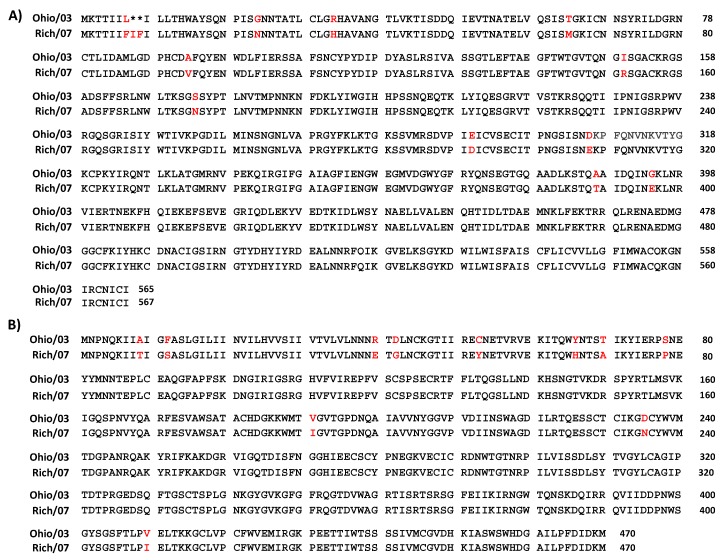
Amino acid sequence alignments of Ohio/03 (top) and Rich/07 (bottom) HA (**A**) and NA (**B**). In red are indicated the amino acid residues that differ between the two viruses present in the EIV bivalent LAIV. Residue numbers are indicated at the right as reference.

**Figure 3:**
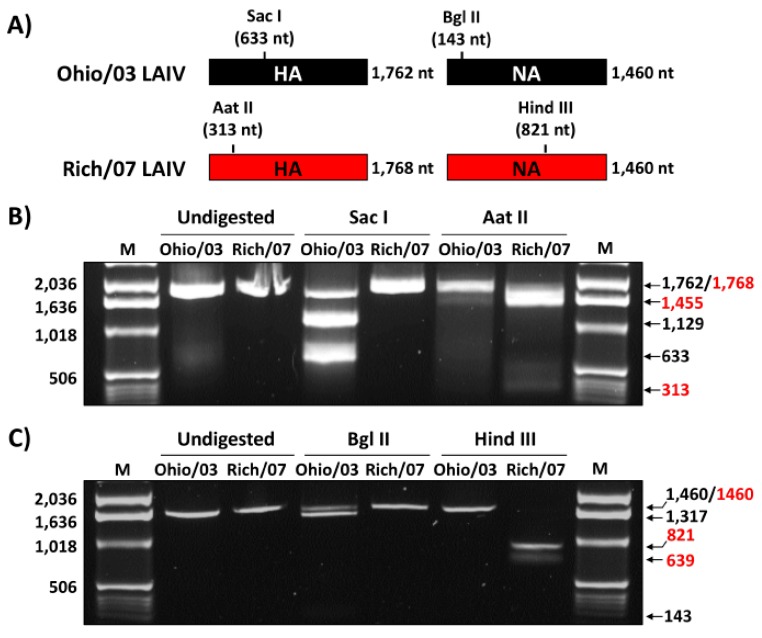
Genotypic characterization of clade 1 Ohio/03 and clade 2 Rich/07 LAIVs: (**A**) Schematic representation of the hemagglutinin (HA; left) and neuraminidase (NA; right) segments from Ohio/03 (black) and Rich/07 (red) LAIVs, indicating the Sac I (Ohio/03) and Aat II (Rich/07); and the Bgl II (Ohio/03) and Hind III (Rich/07) unique restriction sites and their location in the viral HA and NA, respectively. (**B**,**C**) Madin-Darby canine kidney (MDCK) cells (6 well-plate format, 1 × 10^6^ cells/well) were individually infected (MOI 0.1) with Ohio/03 or Rich/07 LAIVs and incubated at 33 °C. At 48 h post-infection (p.i.), total RNA was extracted and the HA (**B**) and NA (**C**) viral segments were amplified by RT-PCR using specific primers. Undigested and Sac I- or Aat II-digested PCR products of HA (**B**) and Bgl II- or Hind III-digested PCR products of NA (**C**) are shown. M: Ladder marker. The nucleotide size of the different bands of the ladder marker is indicated on the left. Nucleotide length of undigested and digested products for Ohio/03 (black) and Rich/07 (red) are indicated on the right.

Because of the similarity in the nucleotide (Supplementary Figures S1 and S2) and amino acid (Figure 2) sequences of Ohio/03 and Rich/07 HA and NA, the identity of the recombinant Ohio/03 and Rich/07 LAIVs was confirmed by RT-PCR and enzyme restriction digestion based on the presence of either Sac I (Ohio/03) or Aat II (Rich/07) restriction sites in the viral HAs (Figure 3A, left); and the presence of either Bgl II (Ohio/03) or Hind III (Rich/07) enzyme restriction sites in the viral NAs (Figure 3A, right). As expected, only the HA from Ohio/03 LAIV was digested with Sac I, generating two segments of 1129 and 633 nucleotides (nt) (Figure 3B). A small fraction remained undigested (1762 nt). In contrast, only the HA from Rich/07 LAIV was digested by Aat II, generating two separated bands of 1455 and 313 nt; while the HA from Ohio/03 remained undigested (Figure 3B). Likewise, Bgl II digestion of Ohio/03 LAIV NA resulted in two bands of 1317 and 143 nt, while the NA from Rich/07 LAIV remained undigested (Figure 3C). Finally, Hind III digestion of Rich/07 LAIV NA resulted in two bands of 821 and 639 nt, while the NA from Ohio/03 remained undigested (Figure 3C). These results demonstrate the identity of both our previously described Ohio/03 and the new Rich/07 LAIVs, which was further confirmed by sequencing of the HA and NA RT-PCR products.

**Figure 4:**
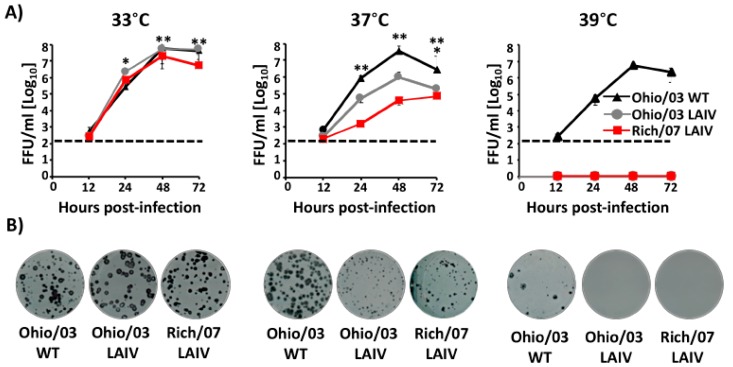
In vitro characterization of Ohio/03 and Rich/07 LAIVs: (**A**) Multicycle growth kinetics: MDCK cells (12-well plate format, 5 × 10^5^ cells/well, triplicates) were infected (multiplicity of infection (MOI) 0.001) with Ohio/03 WT, Ohio/03 LAIV, or Rich/07 LAIV and incubated at 33 °C (left), 37 °C (middle), and 39 °C (right). Tissue culture supernatants from infected cells collected at 12, 24, 48, and 72 h p.i. were used to evaluate the presence of viruses by immunofocus assay (FFU/mL) using an anti-NP monoclonal antibody (HB-65). Data represent the means +/- SDs of the results determined in triplicate wells. Dotted black lines indicate the limit of detection of the assay (200 FFU/mL). Lines below the limit of detection represent non-detected virus. *p* < 0.05: * Ohio/03 WT vs. Ohio/03 LAIV; ** Ohio/03 WT vs. Rich/07 LAIV. (**B**) Plaque assay: MDCK cells (6-well plate format, 1 × 10^6^ cells/well) were infected with Ohio/03 WT, Ohio/03 LAIV, or Rich/07 LAIV and incubated at 33 °C (left), 37 °C (middle), and 39 °C (right). The plaque phenotype was analyzed at 72 h p.i. by immunostaining with the anti-NP monoclonal antibody HB-65.

Next, multicycle growth kinetics (Figure 4A) and the plaque phenotype (Figure 4B) of Ohio/03 and Rich/07 LAIVs were evaluated at different temperatures (33 °C, 37 °C, and 39 °C) in MDCK cells and compared to the Ohio/03 WT. At 33°C, Ohio/03 LAIV and WT grew at similar levels while Rich/07 LAIV titers were slightly reduced (~0.5–1 logs) as compared to Ohio/03 WT (Figure 3A, left). At 37 °C, and as expected, both Ohio/03 and Rich/07 LAIVs viral titers were ~2 logs (Ohio/03) or ~3 logs (Rich/07) lower than those of Ohio/03 WT (Figure 4A, middle). As previously described [61], Ohio/03 LAIV was not detected at 39 °C and the same ts phenotype was observed with Rich/07 LAIV (Figure 4A, right), while Ohio/03 WT efficiently replicated at 39 °C. Likewise, we observed that all three viruses displayed a similar plaque phenotype at 33 °C (Figure 4B, left), while a reduction in plaque sizes was observed with Ohio/03 and Rich/07 LAIVs compared to the Ohio/03 WT at 37 °C (Figure 4B, middle). Notably, only Ohio/03 WT was able to make plaques at 39 °C. No viral plaques at 39 °C were observed in the case of Ohio/03 and Rich/07 LAIVs (Figure 4B, right). These results demonstrate that the Rich/07 LAIV containing HA and NA from the clade 2 Rich/07 WT in the Ohio/03 LAIV clade 1 MDV backbone is only able to replicate efficiently at the permissive temperature of 33 °C but not at the restrictive temperatures of 37 °C and 39 °C.

### 3.2. Safety Profile of the Bivalent EIV LAIV

**Figure 5:**
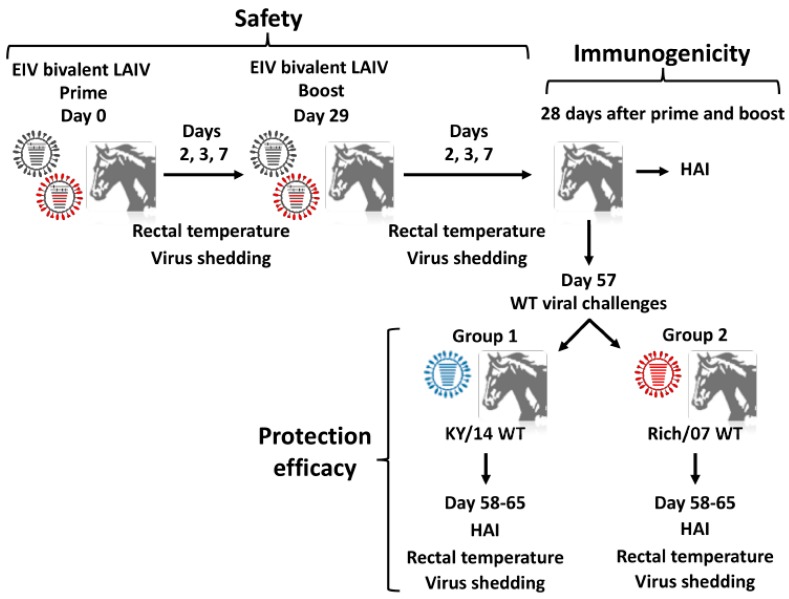
Schematic representation of the vaccination and challenge protocol: One-to-two year-old horses of both sexes (*n* = 12) were randomly separated into group 1 (*n* = 6) and group 2 (*n* = 6) and inoculated by aerosol inhalation with a virus preparation containing 3 × 10^8^ FFU of clade 1 Ohio/03 LAIV and 3 × 10^8^ FFU of clade 2 Rich/07 LAIV using a prime-boost regimen. Individual rectal temperature and viral shedding were measured in each horse before and at days 2, 3, and 7 after each dose (safety). Twenty-eight days after the boost, samples were collected and presence of serum antibodies was assessed by HAI (immunogenicity). Fifty-seven days after prime, vaccinated (*n* = 12) and mock-vaccinated (*n* = 6) horses were randomly separated into group 1 and group 2 and challenged by aerosol inhalation with 5 × 10^7^ EID_50_ of A/equine/Kentucky/2014 WT (KY/14 WT; *n* = 6 vaccinated group 1 and *n* = 3 mock-vaccinated) or 5 × 10^7^ EID_50_ of Rich/07 WT (*n* = 6 vaccinated group 2 and *n* = 3 mock-vaccinated) to assess protection against clade 1 and 2 EIVs, respectively. Rectal temperatures and virus shedding were evaluated (protection efficacy) for eight days after challenge.

To evaluate the safety profile of the bivalent EIV LAIV, horses were inoculated, by aerosol inhalation, with a 1:1 mixture of 3 × 10^8^ FFU of Ohio/03 and Rich/07 LAIVs using a prime-boost regimen (Figure 5). In our previous study, horses that were vaccinated with 3 × 10^8^ FFU of the monovalent Ohio/03 LAIV by intranasal intubation did not develop any clinical signs of infection [61]. Thus, a mixture of an equivalent amount of clade 1 and clade 2 EIV LAIVs was used in this study. Vaccination was followed by close monitoring of clinical signs such as coughing, nasal discharge, respiration, or depression. After first vaccination, all horses remained healthy in appearance (i.e., bright, alert, responsive) with no signs of anorexia/depression. One horse showed a mucopurulent nasal discharge at day 3 p.i. and a slight serous nasal discharge at day 7 p.i. Other three horses showed mucopurulent nasal discharge at day 7 p.i. After the second LAIV dose, none of the vaccinated horses displayed any clinical signs of EIV infection and the behavior of all animals was similar to those of unvaccinated horses or before vaccination.

**Figure 6:**
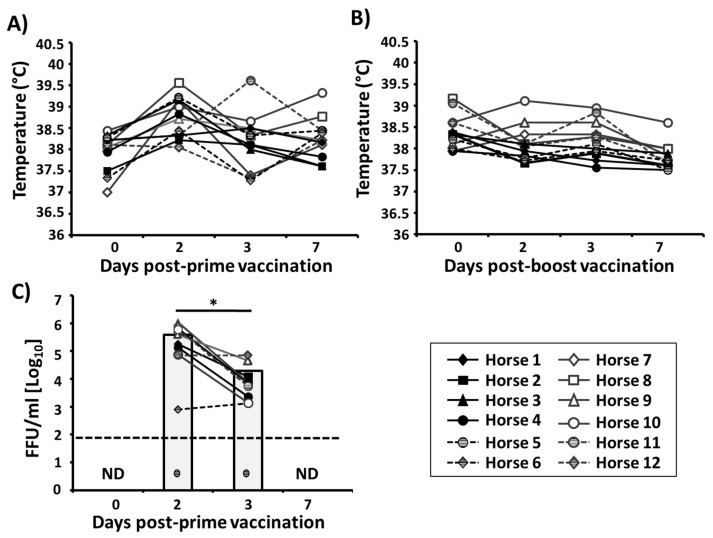
Attenuation of the bivalent EIV LAIV in horses: (**A**,**B**) Graphical representation of the rectal temperatures in vaccinated horses (*n* = 12) before (day 0) and 2, 3, and 7 days after prime (**A**) and boost (**B**) vaccination with the bivalent EIV LAIV. Data represent the rectal temperature of each horse which representative symbol is indicated at the bottom-right. (**C**) Viral titers: Nasopharyngeal swabs from vaccinated horses (*n* = 12) were collected on days 0, 2, 3, and 7 days after prime vaccination. Virus content was determined by immunofocus assay (FFU/mL). Individual results for each horse are represented. Bars indicate the mean of the results determined in the 12 horses. ND, not detected. Dotted black lines indicate the limit of detection (200 FFU/mL). Striped black circles below the limit of detection represent non-detected virus, horse 5. * *p* < 0.05 was considered statistically significant.

In addition, vaccinated horses were closely monitored for changes in rectal temperature and viral shedding. After prime, horses maintained normal mean rectal temperatures as compared with day of vaccination (37.9 °C ± 0.4 day 0 vs. 38.2 °C ± 0.5 day 7) (Figure 6A). The mean rectal temperature was slightly elevated at day 2 p.i. (38.8 °C ± 0.4) but still within normal range, and this elevation was also detected in one of the sentinels (ambient air temperatures that week were ~32 °C). After boost with the bivalent EIV LAIV (Figure 6B), a slight decrease of rectal temperature was observed between days 0 and 7 (38.3 °C ± 0.4 day 0 vs. 37.8 °C ± 0.33 day 7) in vaccinated horses.

Collected nasopharyngeal swabs (one swab per horse per day) during the monitoring period were used to evaluate the presence of EIV LAIVs by immunofocus assay (FFU/mL) (Figure 6C). Virus was detected in 11 out of 12 horses at days 2 and 3 after priming with a decrease in viral titers from day 2 (mean, 3.9 × 10^5^ FFU/mL) to day 3 (mean, 2.12 × 10^4^ FFU/mL), with viral clearance by day 7 post-vaccination (Figure 6C). Importantly, viruses in nasopharyngeal swabs collected after boost were not detected in any of the vaccinated animals, indicating that prime vaccination induced protection at the site of influenza infection. Altogether, these results demonstrated that the bivalent EIV LAIV was able to efficiently replicate in the upper respiratory tract of vaccinated horses, which is necessary for the induction of specific immunity. Replication in the upper respiratory tract did not lead to disease or adverse effects in the vaccinated animals, which remained healthy and were able to spontaneously control viral shedding and infection.

### 3.3. Induction of Serum Antibody Responses

Blood samples were taken at day 0 and 28 days after prime- and boost-vaccination to evaluate the ability of the bivalent EIV LAIV to elicit serum HAI antibodies against Ohio/03, KY/14 and Rich/07 WT viruses. Twenty-eight days after vaccination, low titers of HAI antibodies were detected against all three strains, with the highest rate of seroconversion against Ohio/03 WT (6/12 after prime and 8/12 after boost-vaccination). Only two horses seroconverted against KY/14 WT while one seroconverted against Rich/07 WT after boost doses.

After evaluating the safety of the bivalent EIV LAIV, at 57 days post-V0 the group 1 (*n* = 6) and unvaccinated control (*n* = 3) and sentinel (*n* = 2) horses were challenged with 5 × 10^7^ EID_50_ units of the clade 1KY/14 WT virus, administered by aerosol inhalation. Moreover, at 57 days post-V0, group 2 (*n* = 6) and another group of unvaccinated control horses (*n* = 3) were challenged with the clade 2 Rich/07 WT.

**Figure 7:**
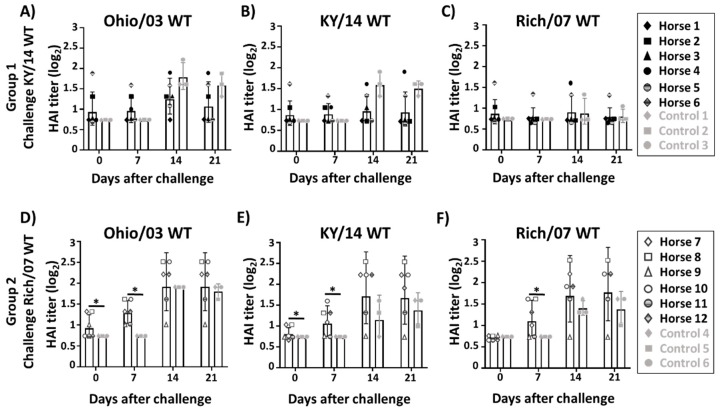
Induction of humoral response by the bivalent EIV LAIV before and after WT viral challenges: Fifty-seven days after prime vaccination, group 1 horses (*n* = 6) were challenged with 5 × 10^7^ EID_50_ of the clade 1 KY/14 WT and group 2 horses (*n* = 6) were challenged with 5 × 10^7^ EID_50_ of the clade 2 Rich/07 WT. Unvaccinated horses (*n* = 3) were used as internal controls in each group. HAI titers against Ohio/03 WT (**A**,**D**), KY/14 WT (**B**,**E**), and Rich/07 WT (**C**,**F**) were determined using sera collected before (day 0) and 7, 14, and 21 days after challenge of group 1 (**A**–**C**) and group 2 (**D**–**F**) horses. Individual HAI titers (log_2_) with sera obtained from vaccinated and control horses are represented as black circles and gray squares, respectively. Bars indicate the geometric mean of the results obtained from the vaccinated or control horses, respectively. An HAI titer of 5 (equivalent to HAI log_2_ of 0.71) was arbitrarily assigned to those data below the limit of detection (<10). * *p* < 0.05 was considered statistically significant.

Blood samples were taken at days 0, 7, 14, and 21 after challenge with clade 1 KY/14 or clade 2 with Rich/07 WT to evaluate anamnestic antibody responses against Ohio/03 (Figure 7A,D), KY/14 (Figure 7B,E), and Rich/07 (Figure 7C,F) WT viruses by HAI. During the first 21 days after challenge of group 1 with KY/14 WT, antibody titers had slightly risen in the vaccinated Ohio/03 WT group 1 (Figure 7A) and the KY/14 WT group 1 (Figure 7B), although non-significant differences were observed between vaccinated and control horses (Figure 7A–C). Importantly, horses challenged with Rich/07 WT (group 2) displayed a marked increment in HAI geometric mean titers (GMT) from day 0 to day 21 for Ohio/03 WT (HAI GMT 0.92 vs. 1.95, *p* < 0.05; Figure 7D), KY/14 WT (HAI GMT 0.80 vs. 1.67, *p* < 0.05; Figure 7E) and Rich/07 WT (HAI titer 0.7 vs. 1.77, *p* < 0.05; Figure 7F). Although no significant differences in HAI GMT were observed at day 21 when comparing vaccinated and control horses challenged with Rich/07 WT, the HAI GMT in vaccinated control horses tended to be higher than in unvaccinated horses against Ohio/03 WT (HAI GMT 1.95 vs.1.79; Figure 7D), KY/14 WT (GMT 1.66 vs. 1.37; Figure 7E), and Rich/07 WT (HAI GMT 1.77 vs.1.37; Figure 7F). Importantly, significant differences between vaccinated and unvaccinated groups were mainly observed at short-term after challenge (day 7) (Figure 7D–F). These results suggest that the bivalent EIV LAIV might allow vaccinated horses to respond faster and better in terms of antibodies than unvaccinated horses during challenge with a homologous virus.

### 3.4. Protection Efficacy of the Bivalent EIV LAIV Against Clade 1 and 2 EIVs

**Figure 8:**
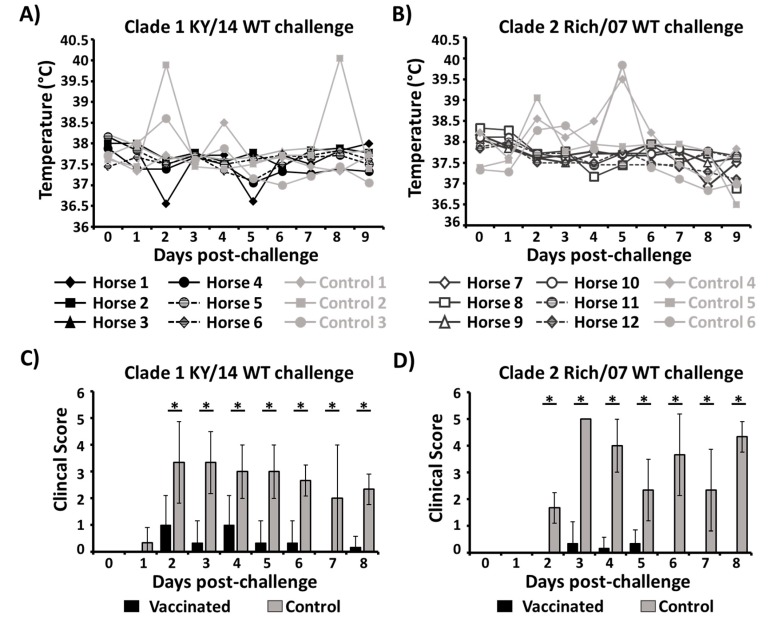
Rectal temperature and clinical scores in vaccinated and control horses after EIV viral challenges: (**A**,**B**) Rectal temperatures: Rectal temperatures were measured daily over 9 days after challenge of group 1 horses with KY/14 WT (**A**) and group 2 horses with Rich/07 WT (**B**). (**C**,**D**) Clinical scores: Clinical signs were recorded over 8 days after challenge of group 1 (**C**) and group 2 (**D**) horses. The clinical signs scoring index is found in Table 1 (maximum clinical score of 6). Data represent the means +/− SDs of the clinical score calculated for vaccinated (black) and control (gray) horses after challenge with KY/14 WT (**C**) and Rich/07 (**D**). * *p* < 0.005 was considered statistically significant.

After challenge with KY/14 (group 1) or Rich/07 (group 2) WT EIVs, horses were monitored daily over eight–nine days for rectal temperatures (Figure 8A,B) and clinical signs (Figure 8C,D). Body temperatures in group 1 vaccinated horses remained within a normal range through the nine days after WT viral challenge (Figure 8A). However, increases in rectal temperature was observed in control horses compared to vaccinated horses at day 2 after challenge (mean increase 0.8 °C). These differences in rectal temperatures between vaccinated and control horses were higher for those horses challenged with Rich/07 WT (Figure 8B). In this case, the mean rectal temperature of control horses increased by 0.98 °C and 1.4 °C at days 2 and 5 post-challenge, respectively. Notably, the three unvaccinated control horses challenged with Rich/07 all displayed pyrexia while all vaccinated horses remained within normal temperature ranges with no significant alterations during the nine days post-challenge.

**Figure 9:**
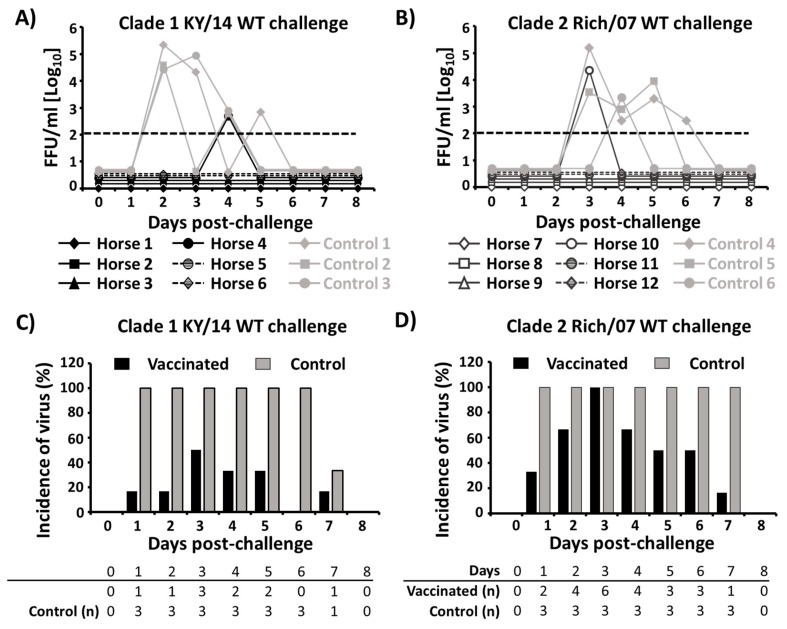
Viral shedding in vaccinated and control horses after EIV WT challenges: (**A**,**B**) Virus titers from nasopharyngeal swabs collected over 8 days after challenge with WT EIV were determined by immunofocus assay (FFU/mL) using an anti-NP monoclonal antibody (HB-65). Data represent individual results from each vaccinated (black) or control (gray) horse in group 1 (**A**) and group 2 (**B**). Dotted black lines represent the limit of detection of the assay (200 FFU/mL). (**C**,**D**) Embryonated chicken eggs were inoculated (100 μL/egg, in triplicate) with nasopharyngeal swabs collected over 8 days after challenge with WT EIVs and presence of challenge virus was evaluated by hemagglutination assay (HA). Horses were considered positive when at least one egg showed progeny virus growth. Bars represent the incidence of positive vaccinated (black) or control (gray) horses in group 1 (**C**) and group 2 (**D**). Number of eggs (*n* = 3) positive for the presence of WT EIV challenge virus are indicated at the bottom.

Clinical signs (Table 1) were monitored for eight days after challenges (Figure 8C,D). Overall, the occurrence of clinical signs in group 1 (Figure 8C) and group 2 (Figure 8D) vaccinated horses was lower and of a shorter duration than in unvaccinated horses. In the KY/14 WT challenge, tachypnea was observed in one unvaccinated control but in none of vaccinates. Nasal discharge was observed in five out of six vaccinates and all three control horses, although it was significantly shorter in vaccinated (2 days ± 0.7) than in control (7.6 days ± 1.1; *p* < 0.001) horses. Importantly, coughing was observed in all three control horses (4.6 days ± 1.5) but not in the vaccinated horses after challenge with KY/14 WT virus. Signs of anorexia were observed in control horses at one–two days after challenge, while normal behavior was observed in vaccinated horses. In the Rich/07 challenge experiment (Figure 8D), all three unvaccinated controls also showed an increase in the time of nasal discharge (4.6 days ± 1.5) compared to vaccinates (one horse, one day only) horses. In addition, only one horse in the vaccinated group was observed to cough from days 3–5 p.i. whereas coughing was observed in all three unvaccinated controls during 5.6 ± 1.15 days. In this group 2, anorexia was only detected in the three unvaccinated control horses beginning at day 3 post-challenge. We did not find a correlation between the HAI titer after vaccination (Figure 7) and the clinical score (Figure 8), suggesting a possible role of mucosal immunity and/or T-cell immunity in protecting from clinical signs after challenge (Supplementary Figure S3). Although horses presenting clinical signs at day 7 were unvaccinated horses, we could not correlate protection from clinical sings with higher HAI titer (Supplementary Figure S3). In addition, viral shedding was undetectable for all horses at day 7 after challenge (Figure 9A,B), independently of the HAI titer.

All six unvaccinated control, but no vaccinated, horses were deemed by an independent and study-blinded veterinarian to require treatment for control of secondary bacterial infections, and were so treated with Ceftiofur crystalline free acid Excede (Zoetis Inc., Parsippany, NJ, USA) beginning in most cases on day 5 post-challenge. Three of the six controls (one in the KY/14 challenge, two in the Rich/07 challenge), but no vaccinated, also received non-steroidal anti-inflammatory treatment with IV flunixin meglumine (Zoetis Inc.) at least once subsequent to challenge.

Virus shedding in nasopharyngeal swabs was also evaluated eight days after challenge by immunofocus assay (Figure 9A,B) and by virus amplification in embryonated chicken eggs (Figure 9C,D). Only one vaccinated horse in group 1 had detectable virus at day 4 p.i. (5 × 10^2^ FFU/mL) while the three control horses were positive for the presence of challenge virus, peaking at day 2 p.i. (Figure 9A). Similarly, the three control horses were positive for virus between days 3–6 p.i. in group 2, while only one vaccinated horse contained virus in nasopharyngeal swabs at day 3 p.i. (2.3 × 10^4^ FFU/mL) (Figure 9B). Importantly, in both horse groups, viral titers found in the nasopharyngeal swabs of the three control horses were higher than those found in the two vaccinated horses (Figure 9A,B). When nasopharyngeal swabs from unvaccinated control horses following KY/14 WT challenge were inoculated in embryonated chicken eggs, an incidence of infection of 100% was found between days 1–6 p.i, while an incidence between 10–50% was found for vaccinated horses, peaking at day 3 p.i. (Figure 9C). Following Rich/07 WT challenge, an incidence of 100% was found in the unvaccinated control group between days 1–7 p.i. while vaccinated horses only reached the 100% at day 3 p.i. and then decreased until day 7 p.i. (Figure 9D). In addition, when we quantified the infectious virus present in the nasopharyngeal swab samples collected at day 2 post-challenge, we observed that viral titers were significantly lower in vaccinated than unvaccinated horses in group 1 (0.33 vs. 5.87 EID_50_, *p* < 0.001) and group 2 (1.90 vs. 6.22 EID_50_, *p* = 0.002) horses. Altogether, these results demonstrate that our bivalent EIV LAIV is able to induce protective but not sterile immune responses against challenge with clade 1 KY/14 WT and clade Rich/07 WT EIVs.

Interestingly, the two sentinel horses co-mingled with group 1 post-vaccination displayed opposite responses to challenge. One showed temperature and clinical profiles very similar to the group 1 vaccinates following challenge, and no virus was detected in nasopharyngeal swabs by FFU assay. The other (observed in pasture to be a less sociable horse) displayed pyrexia and nasal discharge similar to the unvaccinated controls, similarly required antibiotic/anti-inflammatory treatments, and shed detectable virus on day 2 after challenge. This suggests the likelihood that vaccine virus had been spread in the one case but not the other.

## 4. Discussion

Equine influenza caused by H3N8 EIV is one of the major infectious respiratory diseases of horses [2]. This virus can spread through the air reportedly up to 1–2 km and be transmitted via infectious aerosols [75]. Horses are especially susceptible to EIV infection when they are transported because of the stress of travel and exposure to other horses in a closed environment. The worldwide transport of horses by air and the congregation of horses at equestrian events facilitates virus spread that causes important economic losses [24,76] and introduction of EIV to EIV-free zones around the world [77]. Equine influenza surveillance, restricted movement and traffic, proper quarantine practices, and isolation of infected animals are important measures to contain EIV [30]. Apart from those preventive measures, vaccination is the best prophylactic strategy to control EIV infections in horses [46,47].

Despite prevention efforts made by owners and veterinary practitioners, frequent outbreaks have occurred worldwide with high morbidity in susceptible horse populations [78,79,80]. In 2018–2019, equine influenza outbreaks were reported in South America [81], the US, Europe (United Kingdom, France, Belgium, the Netherlands, and Germany), Nigeria, and South America, including in vaccinated horses, which support claims of inadequate vaccine(s) effectiveness [58]. Different reasons have been proposed for explaining this: improper vaccination schedules, the continued evolution of the EIV genome, and the use of outdated virus strains in the EIV vaccines [27,31]. Thus, continuous surveillance, virus strain characterization, and updating of vaccines with recent circulating strains are urgently needed to control equine influenza in the horse population. Although it has been shown that some cross-reactivity could exist between clade 1 and clade 2 strains [82], cross-reactive antibodies have shown to be less effective than strain-specific antibodies in reducing virus shedding after EIV infection [83]. Since 2010, the OIE has recommended the inclusion of representative clade 1 (A/equine/South Africa/04/2003-like or A/equine/Ohio/2003- like viruses) and clade 2 (represented by A/equine/ Richmond/1/2007-like viruses) strains of the Florida sublineage into equine influenza vaccines. Although recent IIV have been updated to include representative strains of the clades 1 and 2 Florida sublineage, the EIV LAIV Flu Avert I.N. only contains an outdated strain (A/equine/Kentucky/1/1991) that pre-dates both Florida clades. The mismatch between the EIV strain in Flu Avert I.N. and currently circulating strains seems likely to result in inadequate protection of vaccinated horses [27,84,85].

To overcome this limitation, we have generated, for the first time, a bivalent EIV LAIV based on the use of reverse genetics approaches and the inclusion of two representative strains of the clade 1 and clade 2 EIVs of the Florida sublineage recommended by the OIE. Our goal was to evaluate the safety and protection efficacy against viral challenge in its natural host, the horse. The bivalent EIV LAIV was produced by blending our previously generated clade 1 Ohio/03 LAIV [61] with a newly developed recombinant clade 2 Rich/07 LAIV, containing the HA and NA from Rich/07 WT and the six internal segments from Ohio/03 LAIV. Although an unattenuated vaccine group was not included for comparison in horses, our bivalent EIV LAIV was safe, with only minor episodes of nasal discharge and an absence of severe adverse effects after a prime-boost regimen. We anticipate that the concentration/purification steps of a commercial production will eliminate the need for nebulization to administer the vaccine, and with that eliminate even the mild post-vaccination effects we observed. Our bivalent EIV LAIV was detected in the nasal swabs of all vaccinated horses, suggesting the ability of the LAIV to replicate in the upper respiratory tract, which is needed for an effective induction of EIV-specific immune responses in the mucosa. Importantly, virus shedding occurred for less than a week, as has been shown for other EIV LAIVs [35,61]. We observed evidences of viral spread from vaccinated to one unvaccinated horse, which is still consistent with previous results that showed that Flu Avert I.N. had spontaneous transmissibility [54]. This fact should not be a cause of concern as that horse showed no clinical signs. It is possible that our nebulization method by face mask has left virus residues on noses of vaccinated horses, resulting in transmission to the exposed sentinel. This issue of shed-spread does need to be examined in future studies.

As previously reported in the literature [55,72], circulating antibody responses in naïve horses following vaccination were low and this is believed to be a consequence of the route of administration. In contrast to naturally exposed horses, it is well known that after vaccination with LAIV the serum antibody level does not correlate with protection [54,55,72]. In fact, Flu Avert I.N. conferred complete clinical protection with little to no detectable serum antibody [54]. This fact suggests that a circulating antibody-independent immunity could be responsible of the protective response [36]. Although there is still little information about the role of CTL in protection against EIV, some studies have suggested that CTL could have an important role in protection in the absence of detectable antibody [36].

In this study, anamnestic antibody responses tended to be higher in vaccinated horses as compared to unvaccinated animals after challenge with either clade 1 or clade 2 Florida sublineage EIVs at early times after challenges (day 7). It is possible that HAI titer are not a good marker to determine protection and future research should be done to measure mucosal immune response. To overcome this limitation, we included other relevant data such as clinical presentation and shedding. The rectal temperatures of all vaccinated horses remained within a normal range with an absence of fever after challenge. In addition, clinical scores of infection were lower, shorter in duration, and less severe in vaccinated horses as compared to unvaccinated horses. None of the vaccinated but all of the control horses were, upon independent veterinary examination, deemed to require antibiotic treatment. Importantly, the reduced coughing seen in vaccinated horses is likely to minimize the risk of EIV dissemination. Notably, unvaccinated horses displayed a higher rate and duration of virus shedding than vaccinated horses. Sterilizing immunity was not conferred by our bivalent LAIV suggesting that a higher vaccination dose or a second boost dose could be suitable for future studies in order to achieve the highest level of immunity. Lack of sterile immunity was also observed in our previous study and even in the case of Flu Avert I.N. [54,61]. In the present study, we observed that our LAIV protected better against heterologous KY/14 WT than against homologous Rich/07 WT. The cross-protection conferred against heterologous KY/14 WT EIV is likely due to the induction of a T cellular response by the internal proteins belonging to Ohio/03 LAIV rather than antibody responses.

Our LAIV meets the requirements for licensing of an EIV vaccine [86] because it follows the OIE recommendation of including representative clade 1 and 2 strains of the Florida sublineage of EIVs, has a safe profile in horses, induces a serological response against viruses present in the vaccine, and confers protection against challenge with both clades 1 and 2 EIVs of the Florida sublineage that are currently circulating in horses. Importantly, since our bivalent EIV LAIV is based on the use of reverse genetics approaches, it would be easy to update against EIVs of epidemiological relevance or newly introduced EIV strains in the horse population. It must be mentioned that EIV has crossed the species barrier to infect other animal species such as dogs, camels, and pigs, and could also potentially pose a threat to humans [87,88,89,90,91]. A better control of EIV in its natural host by using more efficient vaccines like this EIV bivalent LAIV could also prevent the potential infection of other species, including humans.

## Figures and Tables

**Table 1:** Clinical signs scoring index.

Clinical Sign	Degree	Score
Respiration Rate	Normal < 36/min	0
	Abnormal (dyspnea/tachypnea) > 36/min	1
Nasal discharge	No discharge	0
	Abnormal serous	1
	Abnormal mucopurulent	2
Coughing	No coughing	0
	Coughed once	1
	Coughed twice	2
Anorexia	Non	0
	Yes	1

## Supplementary Materials

The following are available online at https://www.mdpi.com/1999-4915/11/10/933/s1, Figure S1: Nucleotide alignment of clade 1 Ohio/03 and clade 2 Rich/07 HA, Figure S2: Nucleotide alignment of clade 1 Ohio/03 and clade 2 Rich/07 NA and Figure S3: Correlation between HAI titer and Clinical Score.
