# Supplementary material for: A Bivalent Live-Attenuated Vaccine for the Prevention of Equine Influenza Virus
Source: Viruses. 2019 Oct 11;11(10):933. doi: 10.3390/v11100933 (PMC6832603; doi:10.3390/v11100933)

Supplementary Figure 1. Nucleotide alignment of clade 1 Ohio/03 and clade 2 Rich/07 HA proteins: In red are indicated the amino acid residues that differ between the two viruses present in the EIV bivalent LAIV. Residue numbers are indicated at the right as reference.

**Ohio/03**	ATGAAGACAA	CCATTATTTT	G******ATA	CTACTGACCC	ATTGGGCCTA	CAGTCAAAAC	CCAATCAGTG	GCAACAACAC	74
**Rich/07**	ATGAAGACAA	CCATTATTTT	TATTTTTATA	CTACTGACCC	ATTGGGCCTA	CAGTCAAAAC	CCAATCAGTA	ACAACAACAC	80
**Ohio/03**	AGCCACATTG	TGTCTGGGAC	GCCATGCAGT	AGCAAATGGA	ACATTGGTAA	AAACAATAAG	TGATGATCAA	ATTGAGGTGA	154
**Rich/07**	AGCCACATTG	TGTCTGGGAC	ACCATGCAGT	AGCAAATGGA	ACATTAGTAA	AAACAATAAG	TGATGATCAA	ATTGAGGTGA	160
**Ohio/03**	CAAATGCTAC	AGAATTAGTT	CAGAGCATTT	CAACGGGGAA	AATATGCAAC	AACTCATATA	GAATTCTAGA	TGGAAGAAAT	234
**Rich/07**	CAAATGCTAC	AGAATTAGTT	CAGAGCATTT	CAATGGGGAA	AATATGCAAC	AACTCATATA	GAATTCTAGA	TGGAAGAAAT	240
**Ohio/03**	TGCACATTAA	TAGATGCAAT	GCTAGGAGAC	CCCCACTGTG	ACGCCTTTCA	GTATGAGAAT	TGGGACCTCT	TTATAGAAAG	314
**Rich/07**	TGCACATTAA	TAGATGCAAT	GCTAGGAGAC	CCCCACTGTG	ACGTCTTTCA	GTATGAGAAT	TGGGACCTCT	TTATAGAAAG	320
**Ohio/03**	AAGCAGCGCT	TTCAGCAATT	GCTACCCATA	TGACATCCCT	GACTATGCAT	CGCTCCGATC	CATTGTAGCA	TCCTCAGGAA	394
**Rich/07**	AAGCAGCGCT	TTCAGCAATT	GCTACCCATA	TGACATCCCT	GACTATGCAT	CGCTCCGATC	AATTGTAGCA	TCCTCAGGAA	400
**Ohio/03**	CATTGGAATT	CACAGCAGAG	GGATTCACAT	GGACAGGTGT	CACTCAAAAC	GGAATAAGTG	GAGCCTGCAA	AAGGGGATCA	474
**Rich/07**	CATTGGAATT	CACAGCAGAG	GGATTCACAT	GGACAGGTGT	CACTCAAAAC	GGAAGAAGTG	GAGCCTGCAA	AAGGGGATCA	480
**Ohio/03**	GCCGATAGTT	TCTTTAGCCG	ACTGAATTGG	CTAACAAAAT	CTGGAAGCTC	TTACCCCACA	TTGAATGTGA	CAATGCCTAA	554
**Rich/07**	GCCGATAGTT	TCTTTAGCCG	ACTGAATTGG	CTAACAAAAT	CTGGAAACTC	TTATCCCACA	TTGAATGTGA	CAATGCCTAA	560
**Ohio/03**	CAATAAAAAT	TTCGACAAGC	TATACATCTG	GGGGATTCAT	CACCCGAGCT	CAAATCAAGA	GCAGACAAAA	TTGTACATCC	634
**Rich/07**	CAATAAAAAT	TTCGACAAGC	TATACATCTG	GGGGATTCAT	CACCCGAGTT	CAAATCAAGA	GCAGACAAAA	TTGTATATCC	640
**Ohio/03**	AAGAATCAGG	ACGAGTAACA	GTCTCAACAA	AAAGAAGTCA	ACAAACAATA	ATCCCTAACA	TCGGATCTAG	ACCGTGGGTC	714
**Rich/07**	AAGAATCAGG	ACGAGTAACA	GTCTCAACAA	AAAGAAGTCA	ACAAACAATA	ATCCCTAACA	TCGGATCTAG	ACCGTGGGTC	720
**Ohio/03**	AGAGGTCAAT	CAGGCAGGAT	AAGCATATAC	TGGACCATTG	TAAAACCTGG	AGATATCCTA	ATGATAAACA	GTAATGGCAA	794
**Rich/07**	AGAGGTCAAT	CAGGCAGGAT	AAGCATATAC	TGGACCATTG	TAAAACCTGG	AGATATCCTA	ATGATAAACA	GTAATGGCAA	800
**Ohio/03**	CTTAGTTGCA	CCGCGGGGAT	ATTTTAAATT	GAAAACAGGG	AAAAGCTCTG	TAATGAGATC	AGATGTACCC	ATAGAAATTT	874
**Rich/07**	CTTAGTTGCA	CCGCGGGGAT	ATTTTAAATT	GAAAACAGGG	AAAAGCTCTG	TAATGAGATC	AGATGTACCC	ATAGACATTT	880
**Ohio/03**	GTGTGTCTGA	ATGTATTACA	CCAAATGGAA	GCATCTCCAA	CGACAAGCCA	TTCCAAAATG	TGAACAAAGT	TACATATGGA	954
**Rich/07**	GTGTGTCTGA	ATGTATTACA	CCAAATGGAA	GCATCTCCAA	CGAAAAGCCA	TTCCAAAATG	TAAACAAAGT	TACATATGGA	960
**Ohio/03**	AAATGCCCCA	AGTATATCAG	GCAAAACACT	TTAAAGCTGG	CCACTGGGAT	GAGGAATGTA	CCAGAAAAGC	AAATCAGAGG	1,034
**Rich/07**	AAATGCCCCA	AATATATCAG	GCAAAACACT	TTAAAGTTGG	CCACTGGAAT	GAGAAATGTA	CCAGAAAAGC	AAATCAGAGG	1,040
**Ohio/03**	AATCTTCGGA	GCAATAGCGG	GATTCATCGA	AAACGGCTGG	GAAGGAATGG	TTGATGGGTG	GTATGGGTTC	CGATATCAAA	1,114
**Rich/07**	AATCTTTGGA	GCAATAGCGG	GATTCATCGA	AAACGGCTGG	GAAGGAATGG	TTGATGGGTG	GTATGGGTTC	CGATACCAAA	1,120
**Ohio/03**	ACTCTGAAGG	AACAGGGCAA	GCTGCAGATC	TAAAGAGCAC	TCAAGCAGCC	ATCGACCAGA	TTAATGGAAA	GTTAAACAGA	1,194
**Rich/07**	ACTCTGAAGG	AACAGGACAA	GCTGCAGATC	TAAAGAGCAC	TCAAACAGCC	ATCGACCAGA	TTAATGAAAA	GTTAAACAGA	1,200
**Ohio/03**	GTGATTGAAA	GAACCAATGA	GAAATTCCAT	CAAATAGAGA	AGGAATTCTC	AGAAGTAGAA	GGAAGAATTC	AGGACTTGGA	1,274
**Rich/07**	GTGATTGAAA	GAACCAATGA	AAAATTCCAT	CAGATAGAGA	AGGAATTCTC	AGAAGTAGAA	GGAAGAATTC	AGGACTTGGA	1,280
**Ohio/03**	GAAATATGTA	GAAGACACCA	AAATAGACCT	ATGGTCCTAC	AATGCAGAAT	TGCTGGTGGC	TCTAGAAAAT	CAACATACAA	1,354
**Rich/07**	GAAATATGTG	GAAGACACCA	AAATAGACCT	ATGGTCCTAC	AATGCAGAAT	TGCTGGTGGC	TCTAGAAAAT	CAACATACAA	1,360
**Ohio/03**	TTGACTTAAC	AGATGCAGAA	ATGAATAAAT	TATTTGAGAA	GACTAGACGC	CAGTTAAGAG	AAAACGCAGA	AGACATGGGA	1,434
**Rich/07**	TTGACTTAAC	AGATGCAGAA	ATGAATAAAT	TATTCGAGAA	GACTAGACGC	CAGTTAAGAG	AAAACGCAGA	AGACATGGGA	1,440
**Ohio/03**	GGTGGATGTT	TCAAGATTTA	CCACAAATGT	GATAATGCAT	GCATTGGATC	AATAAGAAAT	GGGACATATG	ACCATTACAT	1,514
**Rich/07**	GGTGGATGTT	TCAAGATTTA	CCACAAATGT	GATAATGCAT	GCATTGGATC	AATAAGAAAT	GGGACATATG	ACCATTACAT	1,520
**Ohio/03**	ATACAGAGAT	GAAGCATTAA	ACAACCGATT	TCAGATCAAA	GGTGTAGAGT	TGAAATCAGG	CTACAAAGAT	TGGATACTGT	1,594
**Rich/07**	ATACAGAGAT	GAAGCATTAA	ACAACCGATT	TCAAATCAAA	GGTGTTGAGT	TGAAATCAGG	CTACAAAGAT	TGGATACTGT	1,600
**Ohio/03**	GGATTTCATT	CGCCATATCA	TGCTTCTTAA	TTTGCGTTGT	TCTATTGGGT	TTCATTATGT	GGGCTTGCCA	AAAAGGCAAC	1,674
**Rich/07**	GGATTTTATT	CGCCATATCA	TGCTTCTTAA	TTTGCGTTGT	TCTATTGGGT	TTTATTATGT	GGGCTTGCCA	AAAAGGCAAC	1,680
**Ohio/03**	ATCAGATGCA	ACATTTGCAT	TTGA	1,698					
**Rich/07**	ATCAGATGCA	ACATTTGCAT	TTGA	1,708					

Supplementary Figure 2. Nucleotide alignment of clade 1 Ohio/03 and clade 2 Rich/07 NA proteins: In red are indicated the amino acid residues that differ between the two viruses present in the EIV bivalent LAIV. Residue numbers are indicated at the right as reference.

**Ohio/03**	ATGAATCCAA	ATCAAAAGAT	AATAGCAATT	GGATTTGCAT	CATTGGGGAT	ATTAATCATT	AATGTCATTC	TCCATGTAGT	**80**
**Rich/07**	ATGAATCCAA	ATCAAAAGAT	AATAACAATT	GGATCTGCAT	CATTGGGGAT	ATTAATCATT	AACGTCATTC	TCCATGTAGT	
**Ohio/03**	CAGCATTATA	GTAACAGTAC	TGGTCCTCAA	TAACAATGAA	ACAGATCTGA	ACTGCAAAGG	GACGATCATA	AGAGAGTGCA	**160**
**Rich/07**	CAGCATTATA	GTAACAGTAC	TGGTCCTCAA	TAACAATAGA	ACAGGTCTGA	ACTGCAAAGG	GACGATCATA	AGAGAGTACA	
**Ohio/03**	ATGAAACAGT	AAGAGTAGAA	AAAATTACTC	AATGGTATAA	TACCAGTACA	ATTAAGTACA	TAGAGAGACC	TTCAAATGAA	**240**
**Rich/07**	ATGAAACAGT	AAGAGTAGAA	AAAATTACTC	AATGGCATAA	TACCAGTGCA	ATTAAGTACA	TAGAGAGACC	TCCAAATGAA	
**Ohio/03**	TACTACATGA	ACAACACTGA	ACTACTTTGT	GAGGCCCAAG	GCTTTGCACC	ATTTTCCAAA	GATAATGGAA	TACGAATTGG	**320**
**Rich/07**	TACTACATGA	ACAACACCGA	ACCACTTTGT	GAGGCCCAAG	GCTTTGCACC	ATTTTCCAAA	GATAATGGAA	TACGAATTGG	
**Ohio/03**	GTCGAGAGGC	CATGTTTTTG	TGATAAGAGA	ACCTTTTGTA	TCATGTTCGC	CCTCAGAATG	TAGAACCTTT	TTCCTCACAC	**400**
**Rich/07**	GTCGAGAGGC	CATGTTTTTG	TGATAAGAGA	ACCTTTTGTA	TCATGTTCGC	CCTCAGAATG	TAGAACCTTT	TTCCTCACAC	
**Ohio/03**	AGGGCTCATT	ACTCAATGAC	AAACATTCTA	ACGGCACAGT	AAAGGACCGA	AGTCCGTATA	GGACTTTGAT	GAGTGTCAGA	**480**
**Rich/07**	AGGGCTCATT	ACTCAATGAC	AAACATTCTA	ACGGCACAGT	AAAGGATCGA	AGTCCATATA	GGACTTTGAT	GAGTGTCAAA	
**Ohio/03**	ATAGGGCAAT	CACCTAATGT	ATATCAAGCT	AGGTTTGAAT	CGGTAGCATG	GTCAGCAACA	GCATGCCATG	ATGGAAAAAA	**560**
**Rich/07**	ATAGGGCAAT	CACCTAATGT	GTATCAAGCT	AGGTTTGAAT	CGGTGGCATG	GTCAGCAACA	GCATGCCATG	ATGGAAAAAA	
**Ohio/03**	ATGGATGACA	GTTGGAGTCA	CAGGGCCCGA	CAATCAAGCA	ATTGCAGTAG	TGAACTATGG	AGGTGTTCCG	GTTGATATTA	**640**
**Rich/07**	ATGGATGACA	ATTGGAGTCA	CAGGGCCCGA	CAATCAAGCA	ATTGCAGTAG	TGAACTATGG	GGGTGTTCCG	GTTGATATTA	
**Ohio/03**	TTAATTCATG	GGCAGGGGAT	ATTTTAAGAA	CCCAAGAATC	ATCATGCACC	TGCATTAAAG	GAGACTGTTA	TTGGGTAATG	**720**
**Rich/07**	TTAATTCATG	GGCAGGGGAC	ATCTTAAGAA	CCCAAGAATC	ATCATGCACC	TGCATTAAAG	GAAACTGTTA	TTGGGTAATG	
**Ohio/03**	ACTGATGGAC	CGGCAAATAG	GCAAGCTAAA	TATAGAATAT	TCAAAGCAAA	AGATGGAAGA	GTAATTGGAC	AGACTGATAT	**800**
**Rich/07**	ACTGATGGAC	CGGCAAATAG	GCAAGCTAAA	TATAGGATAT	TCAAAGCAAA	AGATGGAAGA	GTAATTGGAC	AGACTGATAT	
**Ohio/03**	AAGTTTCAAT	GGGGGACACA	TAGAGGAGTG	TTCTTGTTAC	CCCAATGAAG	GGAAGGTGGA	ATGCATATGC	AGGGACAATT	**880**
**Rich/07**	AAGCTTCAAT	GGGGGACACA	TAGAGGAGTG	TTCTTGTTAC	CCCAATGAAG	GGAAGGTGGA	ATGCATATGC	AGGGACAATT	
**Ohio/03**	GGACTGGAAC	AAATAGACCA	ATTCTGGTAA	TATCTTCTGA	TCTATCGTAC	ACAGTTGGA	TATTTGTGTGC	TGGCATTCCC	**960**
**Rich/07**	GGACTGGAAC	AAATAGACC	AATTCTGGTAA	TATCTTCTGA	TCTATCGTAC	ACAGTTGGA	TATTTGTGTGC	TGGCATTCCC	
**Ohio/03**	ACTGACACTC	CTAGGGGAGA	GGATAGTCAA	TTCACAGGCT	CATGTACAAG	TCCTTTGGGA	AATAAAGGAT	ACGGTGTAAA	**1,040**
**Rich/07**	ACTGACACTC	CTAGGGGAGA	GGATAGTCAA	TTCACAGGCT	CATGTACAAG	TCCTTTGGGA	AATAAAGGAT	ACGGTGTAAA	
**Ohio/03**	AGGTTTCGGG	TTTCGACAAG	GAACTGACGT	ATGGGCCGGA	AGGACAATTA	GTAGGACTTC	AAGATCAGGA	TTCGAAATAA	**1,120**
**Rich/07**	AGGTTTCGGG	TTTCGACAAG	GAACTGACGT	ATGGGCCGGA	AGGACAATTA	GTAGGACTTC	GAGATCAGGA	TTCGAAATAA	
**Ohio/03**	TAAAAATCAG	GAATGGTTGG	ACACAGAACA	GTAAAGACCA	AATCAGGAGG	CAAGTGATTA	TCGATGACCC	AAATTGGTCA	**1,200**
**Rich/07**	TAAAAATCAG	GAATGGTTGG	ACACAGAACA	GTAAAGACCA	AATCAGGAGG	CAAGTGATTA	TCGATGACCC	AAATTGGTCA	
**Ohio/03**	GGATATAGCG	GTTCTTTCAC	ATTGCCGGTT	GAACTAACAA	AAAAGGGATG	TTTGGTCCCC	TGTTTCTGGG	TTGAAATGAT	**1,280**
**Rich/07**	GGATATAGCG	GTTCTTTCAC	ATTGCCGATT	GAACTAACAA	AAAAGGGATG	TTTGGTCCCC	TGTTTCTGGG	TTGAAATGAT	
**Ohio/03**	TAGAGGTAAA	CCTGAAGAAA	CAACAATATG	GACCTCTAGC	AGCTCCATTG	TGATGTGTGG	AGTAGATCAT	AAAATTGCCA	**1,360**
**Rich/07**	TAGAGGTAAA	CCTGAAGAAA	CAACAATATG	GACCTCTAGC	AGCTCCATTG	TGATGTGTGG	AGTAGATCAT	AAAATTGCCA	
**Ohio/03**	GTTGGTCATG	GCACGATGGA	GCTATTCTTC	CCTTTGACAT	CGATAAGATG	TAA	**1,413**		
**Rich/07**	GTTGGTCATG	GCACGATGGA	GCTATTCTTC	CCTTTGACAT	CGATAAGATG	TAA			

Supplementary Figure 3. Correlation between HAI titers (Figure 7) and clinical scores (Figure 8) at day 7 after challenge with Clade 1 KY/14 (A) and Clade 2 Rich/07 (B) WT viruses.

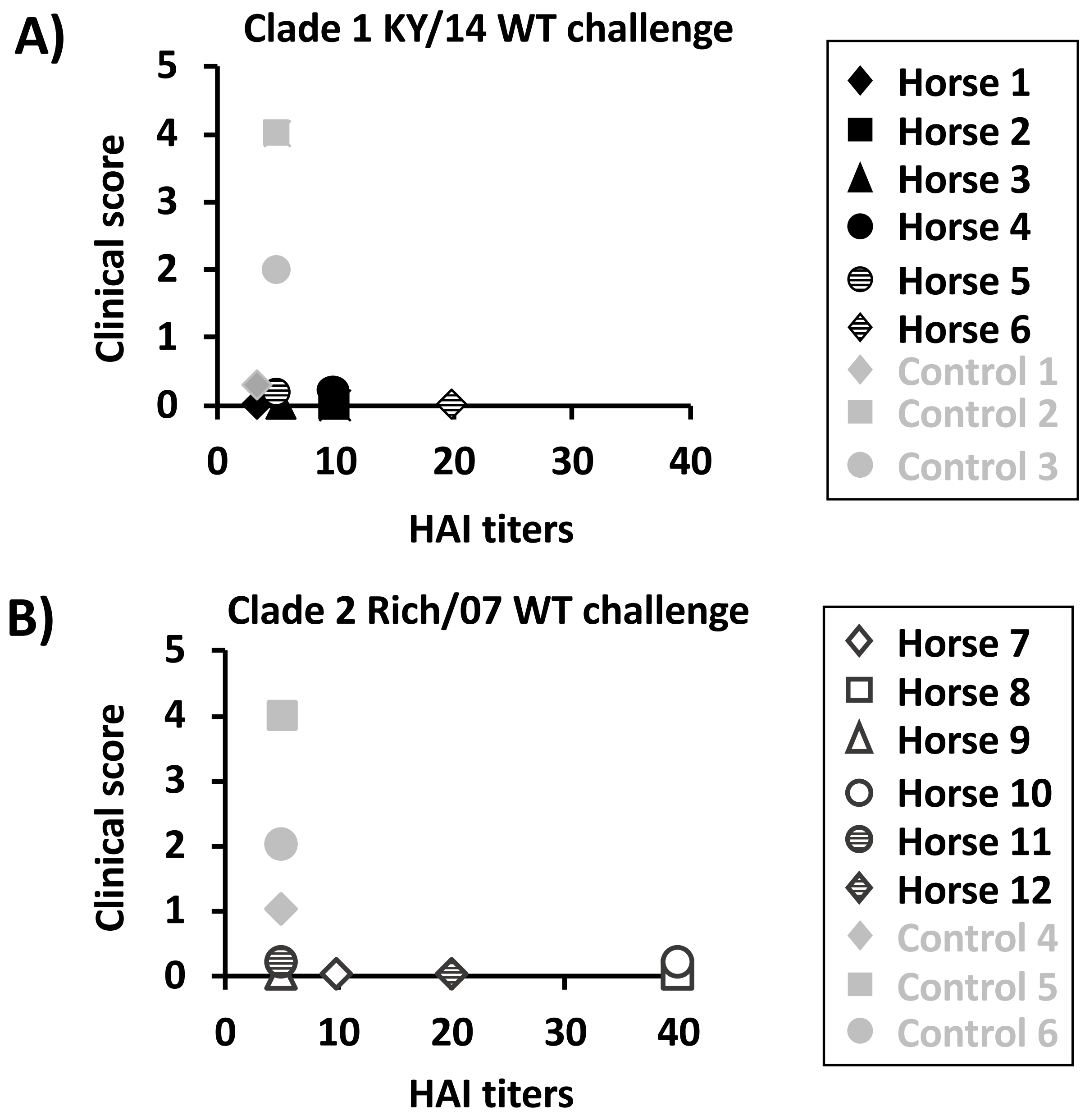

Supplement: Supplementary file 1 [file viruses-11-00933-s001.pdf]
